# Complete Genome of Chronic Bee Paralysis Virus Strain SLO/M92/2010, Detected from *Apis mellifera carnica*

**DOI:** 10.1128/genomeA.00602-17

**Published:** 2017-06-29

**Authors:** Urska Jamnikar-Ciglenecki, Ivan Toplak, Urska Kuhar

**Affiliations:** a Institute of Food Safety, Feed, and Environment, Veterinary Faculty, University of Ljubljana, Ljubljana, Slovenia; b Institute of Microbiology and Parasitology, Veterinary Faculty, University of Ljubljana, Ljubljana, Slovenia

## Abstract

Chronic bee paralysis virus (CBPV) causes an infectious and contagious disease of adult honeybees. Here, we report the complete genome sequence of CBPV strain SLO/M92/2010. This is the first published complete genome of CBPV in *Apis mellifera carnica*, which provides important additional knowledge about the divergence of the CBPV genome.

## GENOME ANNOUNCEMENT

*Apis mellifera carnica* (Carniolan honeybee) is a subspecies of the western honeybee *Apis mellifera* and is an authentic breed in Slovenia. Chronic bee paralysis virus (CBPV) causes infectious and contagious disease of adult honeybees called chronic paralysis (1). Affected bees become flightless and are often found crawling on the ground. Sick bees die within a few days of the onset of symptoms (2–4). CBPV is a positive single-stranded RNA virus but, to date, remains unclassified by the International Committee on Taxonomy of Viruses. It consists of two segments: RNA1, which contains three overlapping open reading frames (ORFs), and RNA2, which contains four overlapping ORFs (5).

The presence of five different honeybee viruses in Slovenia has been monitored within the control program since 2007, and the prevalence of CBPV in affected honeybee colonies has varied from 12.7 to 18.3% (6). The CBPV field strain SLO/M92/2010 reported in this study is a highly pathogenic strain, first detected in Slovenia in 2010, from infected worker bees with symptoms of paralysis (7). Until now, only three studies characterizing the whole genome of CBPV have been published (5, 8, 9), and this is the first report of CBPV isolated from *Apis mellifera carnica*.

Total RNA for next-generation sequencing was extracted with Trizol reagent (Invitrogen, USA). The cDNA was synthesized with the cDNA Synthesis System (Roche, Manheim, Germany) and fragmented with Covaris M220, targeting peak fragment lengths of 400 bp. The library was prepared with the GeneRead DNA Library L Core Kit (Qiagen, Hilden, Germany) and quantified with the GeneRead Library Quant Kit (Qiagen, Hilden, Germany) and the Qubit version 3.0 fluorometer (Thermo Fisher Scientific, USA). Sequencing was performed on the Ion PGM platform. Sequenced reads were quality checked and trimmed using the Ion Torrent Suite version 5.0.5. For CBPV genome assembly, a reference genome with accession numbers KU950353 (segment RNA1) and KU950354 (segment RNA2) was selected from GenBank. Reads were mapped to the reference genome using the Geneious version 9.1.8 software suite (Biomatters Ltd., New Zealand) with default parameters. Consensus sequences of both RNA segments representing the CBPV SLO/M92/2010 genome were constructed. In total, 374,713 reads were mapped to the reference genome with an average depth of 19,448× and an average map length of 308 nucleotides (nt). The CBPV SLO/M92/2010 genome segments RNA1 (3,636 nt) and RNA2 (2,305 nt) were aligned with CBPV genomes deposited in GenBank. The nucleotide identity of the CBPV SLO/M92/2010 strain and CBPV strains deposited in GenBank ranged from 91.8 to 92.8% for the segment RNA1 and from 91.8 to 92.5% for the segment RNA2.

This is the first published complete genome of CBPV detected from *Apis mellifera carnica*. The new complete CBPV genome provides important additional knowledge about the divergence of the CBPV genome.

### Accession number(s).

The complete genome sequences of CBPV strain SLO/M92/2010 have been deposited in GenBank under the accession numbers KY937971 (RNA1) and KY937972 (RNA2).
